# Antifungal Activity of Phlorotannins against Dermatophytes and Yeasts: Approaches to the Mechanism of Action and Influence on *Candida albicans* Virulence Factor

**DOI:** 10.1371/journal.pone.0072203

**Published:** 2013-08-12

**Authors:** Graciliana Lopes, Eugénia Pinto, Paula B. Andrade, Patrícia Valentão

**Affiliations:** 1 REQUIMTE/Laboratório de Farmacognosia, Departamento de Química, Faculdade de Farmácia, Universidade do Porto, Porto, Portugal; 2 CEQUIMED/Laboratório de Microbiologia, Departamento de Ciências Biológicas, Faculdade de Farmácia, Universidade do Porto, Porto, Portugal; Worcester Polytechnic Institute, United States of America

## Abstract

In the last few decades, fungal infections, particularly nosocomial, increased all around the world. This increment stimulated the search for new antifungal agents, especially those derived from nature. Among natural products, those from marine sources have gained prominence in the last years. Purified phlorotannins extracts from three brown seaweeds (*Cystoseira nodicaulis* (Withering) M. Roberts, *Cystoseira usneoides* (Linnaeus) M. Roberts and *Fucus spiralis* Linnaeus) were screened for their antifungal activity against human pathogenic yeast and filamentous fungi. The purified phlorotannins extracts from the studied seaweeds displayed fungistatic and fungicidal activity against yeast and dermatophytes, respectively, pointing to their interest as anti-dermatophyte agent. *C. albicans* ATCC 10231 was the most susceptible among yeast, while *Epidermophyton floccosum* and *Trichophyton rubrum* were the most susceptible among dermatophytes. Since the antifungal mechanism constitutes an important strategy for limiting the emergence of resistance to the commercially available agents, the mechanism of action of purified phlorotannins extracts was approached. *C. nodicaulis* and *C. usneoides* seem to act by affecting the ergosterol composition of the cell membrane of yeast and dermatophyte, respectively. *F. spiralis* influenced the dermatophyte cell wall composition by reducing the levels of chitin. Phlorotannins also seem to affect the respiratory chain function, as all of the studied species significantly increased the activity of mitochondrial dehydrogenases and increased the incorporation of rhodamine 123 by yeast cells. Phlorotannins from *F. spiralis* inhibited the dimorphic transition of *Candida albicans*, leading to the formation of pseudohyphae with diminished capacity to adhere to epithelial cells. This finding is associated with a decrease of *C. albicans* virulence and capacity to invade host cells and can be potentially interesting for combined antifungal therapy, namely for the control of invasive candidiasis.

## Introduction

Resistance to antifungal agents has significantly increased over the past few decades. *Candida albicans* and *Trichophytom rubrum* are among the most common fungal agents, frequently responsible for a variety of infections, ranging from superficial mycoses to life threatening systemic infections [1]. Both yeast and dermatophytes infections can become important causes of morbidity and mortality, especially among immunocompromised patients, with important implications in the health care costs of hospitals and communities [2]. An inevitable consequence of the increased use of antifungal agents in the past decades is the increment of the number and variety of fungal resistance. Hence, considering the emerging multidrug resistance, substantial attention has been focused on natural products with antifungal properties, stimulating the search for therapeutic alternatives [2], [3]. The study of antifungals' mechanism of action constitutes an important strategy for limiting the emergence of resistance to the commercially available agents, as well as to develop safer and more potent compounds in the future.

The cell membrane and cell wall of fungi are the most important targets for antifungal drugs. These physical and chemical barriers are responsible for the communication with the environment and, therefore, have a key role in metabolic processes [1], [4]. Ergosterol is the predominant sterol in fungal cell membranes, responsible for maintaining cell integrity, viability, function and normal growth. The three major groups of antifungal agents in clinical use include azoles, polyenes and allylamines, which owe their antifungal activity to the interaction with ergosterol or to the inhibition of its synthesis [2]. As it happens with the membrane, fungal cell wall is a target for antifungals action. Over the past decades a number of compounds able to affect fungal cell wall has been discovered, being active over the synthesis of chitin and β-glucans, which are essential cell wall components, responsible for fungal structure and normal cell growth. Among them, only echinocandins are commercially available. These compounds are able to inhibit β-glucans synthesis, which are unique compounds among the fungal kingdom [4].

Antifungals can also affect the germ tube formation and adhesion of yeasts, and interact with the respiratory chain processes in mitochondria [5], [6]. The inhibition of the germ tube formation of yeast is considered the mechanism by which several antifungal compounds reduce the microorganism's virulence [6], [7]. By affecting the yeast dimorphic transition, fungistatic compounds reduce microorganism's adhesion to target epithelial cells, decreasing the progression of infection and making it easier to overcome [6].

Mitochondria are present in most eukaryotic cells and comprise the respiratory chain. These organelles play several important roles, including generation and regulation of reactive oxygen species (ROS), calcium (Ca^2+^) homeostasis, regulation of apoptosis and metabolic processes, also being responsible for more than 90% of cellular ATP production [8]. Accordingly, compounds with the capacity to affect mitochondrial respiratory chain can be seen as potential cell growth inhibitors, and capable of trigger cell death [5], [8], [9]. Currently, no antifungal drug owes its primary mechanism of action by affecting mitochondria activity. Neverthelless, besides the primary mechanism of action, some antifungal drugs like azoles and polyenes can act in more than one target, having some effect on mitochondria activity [10], [11].

While the medicinal properties of herbs have been recognized since ancient times, there has been a resurgence of interest in the antimicrobial properties of marine organisms. Seaweeds are particularly attractive, not only for the abundance of substances with industrial interest, but also for the diversity on secondary metabolites with interesting pharmaceutical properties [3], [12]. Among them, phlorotannins, characteristic from Phaeophyta, are particularly interesting because they present important biological activities, without exhibiting toxicity to eukaryotic cells [12]. Although there is some evidence of the antifungal efficacy of brown seaweeds phlorotannins [12], [13], as far as we know there has been no work concerning the mechanism of action of these compounds against fungal organisms. This work aims the evaluation of the mechanism of action of phlorotannins. Three species of Phaeophyta were considered, namely *Cystoseira nodicaulis* (Withering) M. Roberts, *Cystoseira usneoides* (Linnaeus) M. Roberts and *Fucus spiralis* Linnaeus.

## Materials and Methods

### Standards and reagents

Dimethyl sulfoxide (DMSO), trypan blue solution (0.4%), sodium chloride (NaCl), 3-(N-morpholino) propanesulfonic acid (MOPS), N-acetylglucosamine, D-(+)-glucosamine hydrochloride, ergosterol (75%), thiazolyl blue tetrazolium bromide (MTT), rhodamine 123 (RHO), 3-methyl-2-benzothiazolinone hydrazone hydrochloride hydrate, ammonium sulphamate (NH_4_ sulphamate), ferric chloride (FeCl_3_) and curdlan from *Alcaligenes faecalis* were purchased from Sigma-Aldrich (St. Louis, MO, USA). Ethanol, acetone, potassium hydroxide (KOH) and sodium hydroxide (NaOH) were from Panreac (Barcelona, Spain). Sodium nitrite (NaNO_2_), potassium hydrogen sulphate (KHSO_4_), silica gel for thin-layer chromatography 60 G (particle size 5–40 µm), cellulose microcrystalline for thin-layer chromatography, n-hexane, HPLC-grade methanol and acetonitrile were obtained from Merck (Darmstadt, Germany). Fluconazole was kindly provided by Pfizer. Sabouraud dextrose agar (SDA) was purchased from Bio-Mèrieux (Marcy L'Etoile, France). RPMI-1640 broth medium (with L-glutamine, without bicarbonate, and with the pH indicator phenol red) was purchased from Biochrom AG (Berlin, Germany). Dulbelcco's phosphate buffered saline (DPBS) was obtained from Gibco (Invitrogen, Paisley, UK). Yeast nitrogen base was from Difco (New Jersey, USA). Proline and aniline blue water soluble (C_37_H_27_N_3_Na_2_O_9_S_3_) were from Fluka (Buchs, Sankt Gallen, CH). Water was deionised using a Milli-Q water purification system (Millipore, Bedford, MA, USA).

### Seaweeds sampling and extracts preparation

Brown seaweeds used in this work were randomly collected in the coast of Peniche (West Portugal) and taxonomically identified [12]. No specific permissions were required for samples collection, as they do not involve endangered or protected species. Each sample consisted of several individuals in the same stage of development. *C. usneoides* and *C. nodicaulis* were collected in 2009 and *F. spiralis* was collected in 2008. After collection, samples were immediately transported to the laboratory in ice boxes, washed with sea water, frozen and lyophilized in a Labconco 4.5 Freezone apparatus (Kansas City, MO, USA). Thereafter, the dried samples were ground (particle size ≤910 µm) and the purified phlorotannins extracts were prepared as previously reported [12]. Briefly, the powdered material was defatted with hexane and extracted with acetone∶water (7∶3). The extract was purified with cellulose, which was further washed with toluene. Cellulose was then rinsed with acetone∶water (7∶3) to recover the adhered phlorotannins. The filtrate was evaporated, and the dried extract was dissolved in H_2_O∶DMSO (9∶1), prior to antifungal activity assays.

### Fungal organisms

Cultures were obtained from the Laboratory of Microbiology, Faculty of Pharmacy, Porto University (Portugal). The antifungal activity of the purified phlorotannins extracts was evaluated against *Candida*, *Aspergillus* and dermatophyte strains: four clinical strains of *Candida* isolated from recurrent cases of oral candidiasis (*Candida albicans* D1, *C. albicans* D5, *Candida glabrata* D10R and *Candida dubliniensis* CD1), four *Candida* reference strains (*C. albicans* ATCC 10231, *Candida krusei* ATCC 6258, *Candida tropicalis* ATCC 13803 and *Candida parapsilosis* ATCC 90018), three *Aspergillus* strains (*Aspergillus niger* ATCC 16404, *Aspergillus fumigatus* ATCC 46645 and *Aspergillus flavus* F44) and five clinical strains of dermatophytes isolated from nails and skin (*Epidermophyton floccosum* FF9, *Trichophyton rubrum* FF5, *Trichophyton mentagrophytes* FF7, *Microsporum canis* FF1 and *Microsporum gypseum* FF3). *C. krusei* (ATCC 6258) was used as quality control. *C. albicans* ATCC 10231, *C. albicans* D1 and *C. albicans* D5 were used for the germ tube inhibition assay. *C. albicans* ATCC 10231 was used in the adherence assay, for the quantification of ergosterol, 1,3-β-D-glucans and chitin and also for the screening of the respiratory chain function, by the evaluation of the mitochondrial dehydrogenase activity and the mitochondrial membrane potential. *T. rubrum* was used for the ergosterol, 1,3-β-D-glucans and chitin quantification, and *T. rubrum* spores were used for the evaluation of the mitochondrial dehydrogenase activity.

All microorganisms were stored in Sabouraud broth medium with 20% glycerol at −70°C and sub-cultured in SDA before each test, to ensure optimal growth conditions and purity.

### Susceptibility tests

Broth microdilution methods based on the Clinical and Laboratory Standard Institute (CLSI) reference documents M27A-3 and M38-A2 for yeast and filamentous fungi, respectively, with minor modifications, were used to determine minimum inhibitory concentrations (MIC) [14]. Briefly, cell or spore suspensions were prepared from recent cultures of the different strains of fungi on SDA with chloramphenicol and diluted to final inoculum of 10^3^ colony forming units (CFU)/mL with RPMI-1640 broth, buffered to pH 7.0 with MOPS. The MIC of purified phlorotannins extracts was determined by two-fold serial dilution method. Dilutions were prepared in RPMI-1640 broth, starting from 62.5 mg/mL dry matter for each extract. The solutions and cell suspensions in the test medium were then distributed into sterile 96-well plates. Controls were tested along with the samples, with maximum DMSO concentrations not exceeding 2.5% (v/v).The plates were incubated in humid atmosphere, without agitation, at 35°C (for *Candida* spp. and *Aspergillus* spp.) or 25°C (for dermatophytes), during 48 h (for *Candida* spp.), 72 h (for *Aspergillus* spp.) or 5 days (for dermatophytes). MICs were recorded as the lowest concentrations resulting in 100% growth inhibition. Fluconazole MIC for *C. krusei* (ATCC 6258) was determined as quality control, and the result was within the recommended limits (data not shown) [14]. Sterility and growth controls in RPMI-1640 medium alone and with 2.5% of DMSO (v/v) were included.

The minimum lethal concentrations (MLC) of purified phlorotannins extracts was determined after 48 h (for *Candida* spp. and *Aspergillus* spp.) and 96 h (for dermatophytes) of incubation, by removing 20 µL from all wells showing no visible growth to SDA plates. The plates were incubated at 35°C for *Candida* spp. and *Aspergillus* spp. and at 25°C for dermatophytes. The MLC was defined as the lowest concentration showing 100% growth inhibition, resulting from the subculture of MIC plates. All the experiments were performed in duplicate and repeated independently three times.

### Study of *C. albicans* virulence factors

#### Germ tube/dimorphic transition inhibition assay

Germ tube inhibition assay was performed according to Pinto and co-workers [15] with minor modifications. Purified phlorotannins extracts were dissolved in water with 10% DMSO and sterilized by filtration through a 0.22 µm pore size membrane. 250 µL of each extract dilution was added to 250 µL of the yeast suspension to obtain appropriate sub-inhibitory concentrations (1/2–1/256 of the MIC). After 3 h of incubation in NYP medium [N-acetylglucosamine (10^−3^ mol/L), Yeast Nitrogen Base (3.35 g/L) and proline (10^−3^ mol/L)] with NaCl (4.5 g/L, pH 6.7±0.1) at 35°C (with shaking), 100 cells from each sample were counted using a hemocytometer, and the percentage of germ tubes was determined. Germ tubes were considered positive when they were at least as long as the diameter of the blastospore, and negative when showing a constriction at the point of connection to the mother cell, typical for pseudohyphae. The assay was performed in duplicate and repeated independently three times for each *Candida* strain.

#### Adherence to epithelial cells

Cell separation and adherence assays were modified from those proposed by Lima-Neto and co-workers [16], as follows. Yeast cells were grown on SDA for 24 h at 35°C and re-suspended in 2 mL of sterile DPBS (pH 6.8), washed twice by centrifugation (300×g, 5 min. in a Rotofix 32 A Hettich centrifuge) with 2 mL of DPBS and finally re-suspended in NYP (2×10^7^ cells/mL). Epithelial cells were donated by the author *via* soft scraping of the cheek mucous membrane with sterile cotton swabs and gently stirred and washed with DPBS by centrifugation (300×g, 5 min each).

Adherence assays were developed by mixing 1 mL of each suspension in a test tube, followed by incubation in the presence of the test compounds, at 35°C under gentle stirring for 2 h. A control without test compound and a control with epithelial cells pre-treated with the test compound were performed along with the samples. After incubation, two drops of trypan blue solution (0.4%) were added to each tube and the mixture was gently shaken. Ten microliters of the stained suspension were transferred to a Neubauer chamber and examined under light microscopy.

### Mechanism of phlorotannins antifungal action

#### Cell membrane ergosterol

Fungi growth conditions for sterol extraction were performed according to Pinto and co-workers [17] with minor modifications. Cell suspensions (10 µL for yeast and 50 µL for dermathophyte) were inoculated in 5 mL of RPMI-1640 medium containing different concentrations of purified phlorotannins extracts, along with a positive control (with fluconazole) and a negative control (without test extract). Cultures were incubated with shaking at 35°C (for *Candida* species) during 48 h and at 25°C (for dermatophyte) during 5 days.

After the incubation period with purified phlorotannins extracts, total intracellular sterols were extracted by saponification [18]. Briefly, fungal cells were harvested by centrifugation at 300×g for 5 min, washed, dried and weighted. Cell pellets were transferred to sterile borosilicate glass screw-cap tubes and 3 mL of 25% alcoholic KOH solution were added to each tube, followed by a vigorous vortex agitation. Cell suspensions were incubated in a water bath at 85°C during 60 min. After cooling at room temperature, sterols were extracted and the organic phase (n-hexane) was transferred to clean glass tubes and evaporated to dryness under nitrogen. The extracted sterols were redissolved in 0.5 mL of methanol and analysed by HPLC-DAD [19]. Ergosterol quantification was achieved by the absorbance recorded in the chromatograms, relative to the external standard, at 280 nm. All the experiments were repeated independently three times.

#### Cell wall 1,3-β-D-glucans

Cell wall 1,3-β-D-glucans content was determined using the aniline blue assay [20]. Cell suspensions [10 µL for yeast (turbidity adjusted at 0.5 MFA) and 50 µL for dermathophyte (10^6^ spores/mL in NaCl 0.85%)] were inoculated in 10 mL of RPMI-1640 medium containing different concentrations of purified phlorotannins extracts. Positive (with caspofungin) and negative (without test extract) controls were also assayed. Cultures were incubated with shaking at 35°C (for *Candida* species) during 48 h and at 25°C (for dermatophyte) during 5 days. Hyphae and yeast cells were harvested by centrifugation, washed with 0.1 M NaOH and lyophilized overnight. Five milligrams of lyophilized material were used for 1,3-β-D-glucans quantification [20]. Fluorescence (*F*) readings were acquired on a fluorescence reader (Synergy™ HT, Biotek Instruments, Winooski, USA) operated by Gen5 software, with 485/20 nm excitation and 528/20 nm emission wavelength. A standard curve was built using a 1,3-β-D-glucan analog (curdlan). Concentrations of glucans in the samples were calculated as follows: 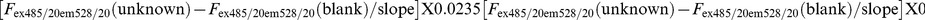



Results (mean ± standard deviation) are expressed as g (curdlan)/100 g (dry microorganism) of three independent assays performed in duplicate.

#### Cell wall chitin

The methodology used for cell wall chitin quantification was based on the protocol proposed by Fortwendel and co-workers [20]. The fungal growth conditions, treatment and tissue harvesting were the same as those proposed for 1,3-β-D-glucans quantification. A standard curve was created using D-(+)-glucosamine hydrochloride as known glucosamine sample. Absorbance (*Abs*) was read at 630 nm on a Multiskan Ascent plate reader (Thermo Electron Corporation, Shanghai, China). Chitin levels were reported as g(glucosamine)/100 g(dry microorganism). Concentrations of glucosamine in the samples were calculated as follows: 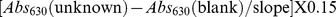



Final results represent the average (± standard deviation) of three independent experiments performed in duplicate.

### Mitochondrial dehydrogenases activity

Mitochondrial dehydrogenase activity was evaluated by the MTT assay [12]. Briefly, *C. albicans* ATCC 10231 cell suspensions were prepared in ampoules containing 2 mL of NaCl 0.85% (api®, Biomérieux, Marcy l'É toile, France), and the turbidity was adjusted to 0.5 MFA. Cell suspension dilutions (1∶50 followed by 1∶20) were prepared with RPMI culture medium. 500 µL of RPMI were added to the same volume of the last dilution, in a 12-well plate, and incubated overnight (18–24 h at 35°C). After the incubation period, cells were carefully homogenised, transferred to eppendorfs and centrifuged at 300×g for 5 min. The supernatant was removed and 1 mL of the extract serial dilutions was added to each eppendorf to obtain the appropriate concentrations (MIC to 1/1024 of the MIC). The mixture was homogenised, transferred to the 12 well plate, and exposed to the purified phlorotannins extract during 1 h at 35°C. For *T. rubrum*, cell suspensions were prepared in 0.85% NaCl solution, and adjusted to obtain 2.0×10^6^ spores/mL. 1 mL of the suspension was centrifuged (300×g, 5 min.), and the supernatant was eliminated. 1 mL of phlorotannins serial dilutions was mixed with the pellet, homogenate and left incubating overnight at 25°C with shaking.

After the exposure time, cell suspensions were centrifuged, the supernatant was removed, and 500 µL of MTT solution (0.5 mg/mL in RPMI, 35°C) were added to the cell pellets and left incubating for 30 minutes at 35°C (for *Candida*) or 25°C (for *T. rubrum*). The insoluble purple formazan product resulting from the conversion of MTT by mitochondrial dehydrogenases of metabolically active cells was then solubilised with 300 µL of DMSO. The extent of the reduction to formazan within the cells was quantified by measuring the absorbance at 510 nm in a Multiskan Ascent plate reader (Thermo Electron Corporation, Shanghai, China). Results from three independent assays performed in duplicate are expressed as the percent change of MTT reduction using the untreated cells as control.

### Mitochondrial membrane potential

The mitochondrial membrane potential was evaluated by the incorporation of the fluorescent dye RHO [21]. Briefly, a suspension of *C. albicans* ATCC 10231 from an overnight culture in SDA was prepared in DPBS and the turbidity adjusted to 2.0 MFA. 880 µL of the cell suspension were mixed with 120 µL of the purified phlorotannins extracts to the desired concentrations (MIC to 1/1024 of the MIC). For controls, purified phlorotannins extracts were replaced by DPBS. After the incubation period (35°C during 30 min), 5 µL of 0.5 mM solution of RHO (in DMSO) were added and incubated again for 10 minutes at 35°C. The exceeding RHO was removed by centrifugation for 5 min. at 300×g, and the fluorescence intensity was determined after resuspending the cell pellet in 1 mL of DPBS.

Fluorescence intensity was determined in a fluorescence microplate reader (Synergy HT, BioTek Instruments, Winooski, USA) equipped with Gen5 software, with excitation wavelength 485/20 nm and emission wavelength 528/20 nm. Results are expressed in% of fluorescence relative to control, for three independent assays performed in duplicate. Sodium azide (an inhibitor of the mitochondrial respiratory chain) at a final concentration of 20 mM was used for all the experiments as control (data not shown).

### Statistical Analysis

Data were analyzed by using GraphPad PRISM software (GraphPad software, San Diego, CA, USA) (version 5.02 for Windows). One-way analysis of variance (ANOVA), using the Dunnett Multiple Comparison test, was carried out on data obtained from three independent assays performed in duplicate for each sample. Levels of statistical significance at *P*<0.05, *P*<0.01 and *P*<0.001 were used.

## Results

### Antifungal activity of phlorotannins purified extracts

The antifungal activity of purified phlorotannins extracts from the studied species is presented in Table 1. The tested extracts displayed antifungal properties against six yeast strains (*C. albicans* ATCC 10231, *C. krusei* ATCC 6258, *C. parapsilosis* ATCC 90018, *C. albicans* D1, *C. albicans* D5 and *C. dubliniensis*), *C. albicans* ATCC 10231 being the most sensitive. It was also possible to determine the MIC_50_ for *C. glabrata* in the tested concentrations (Table 1). With the exception of *M. gypseum*, that was resistant to *C. usneoides*, all of the studied dermatophytes were sensitive to the purified phlorotannins extracts, with fungistatic and fungicidal activity. *T. rubrum* and *E. floccosum* were the most sensitive, being inhibited by all the purified phlorotannins extracts. *C. nodicaulis* presented the lowest MIC and MLC value for both species, followed by *F. spiralis* (Table 1). Under the tested conditions, all of the studied *Aspergillus* species were resistant to the tested extracts at the concentration of 62.5 mg/mL (Table 1). Contrary to *Candida* species, and with the exception of *M. gypseum*, purified phlorotannins extracts presented fungicidal activity against almost all of the studied dermatophyte strains (with MLC = MIC or MLC =  at least one dilution before MIC).

**Table 1:** Activity (MIC/MLC) of purified phlorotannins extracts from brown seaweeds against selected yeast and filamentous fungi^1^.

	Seaweed								
	*C. nodicaulis*			*C. usneoides*			*F. spiralis*		
Strains	MIC^1^	MIC_50_ 1	MLC^1^	MIC^1^	MIC_50_ 1	MLC^1^	MIC^1^	MIC_50_ 1	MLC^1^
***Yeast***									
**ATCC**									
* C. albicans* ATCC 10231	15.6	–	>62.5	31.3	–	>62.5	31.3	–	>62.5
* C. krusei* ATCC 6258	31.3	–	>62.5	31.3	–	>62.5	>62.5	–	–
* C. tropicalis* ATCC 13803	>62.5	–	–	>62.5	–	–	>62.5	–	–
* C. parapsilosis* ATCC 90018	62.5	–	>62.5	62.5	–	>62.5	>62.5	–	–
**Clinical**									
* C. albicans* D1	62.5	–	>62.5	>62.5	62.5	–	>62.5	–	–
* C. albicans* D5	62.5	31.3	>62.5	>62.5	62.5	>62.5	>62.5	–	–
* C. glabrata* D10R	>62.5	2.0	–	>62.5	–	–	>62.5	–	–
* C. dubliniensis* CD1	62.5	31.3	>62.5	62.5	–	>62.5	>62.5	–	–
***Filamentous fungi***									
**Dermatophytes**									
* E. floccosum* FF9	3.9	–	7.8	15.6	–	15.6	7.8	–	7.8
* T. rubrum* FF5	3.9	–	7.8	7.8	–	31.3	3.9	–	31.3
* T. mentagrophytes* FF7	7.8	–	7.8	31.3	–	31.3	15.6	–	15.6
* M. canis* FF1	31.3	–	31.3	31.3	–	>62.5	15.6	–	15.6
* M. gypseum* FF3	31.3	–	>62.5	>62.5	–	–	31.3	–	>62.5
***Aspergillus***									
* A. flavus* F44	>62.5	–	–	>62.5	–	–	>62.5	–	–
* A. fumigatus* ATCC 46645	>62.5	–	–	>62.5	–	–	>62.5	–	–
* A. niger* ATCC 16404	>62.5	–	–	>62.5	–	–	>62.5	–	–

1 MIC, MIC_50_ and MLC were determined by a microdilution method and expressed in mg/mL (dry matter).

“–”Not determined.

### Study of *C. albicans* virulence factors

#### Effect on the dimorphic transition and adherence to epithelial cells of *C. albicans*

**Figure 1:**
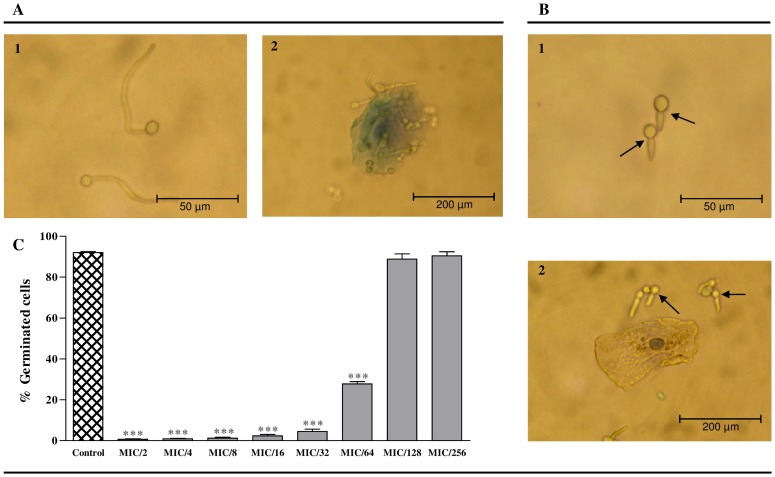
Effect of purified phlorotannins extracts from *F. spiralis* in the dimorphic transition of *C. albicans* ATCC 10231 (Untreated control cells - A1; cells treated with extract at MIC/32 – B1), in the adherence of the yeast to the epithelial cells (Untreated control cells – A2; cells treated with extract at MIC/32 - B2) and in the germ tube formation (C). Arrows show a constriction resulting from an incomplete budding, where the bud remains attached to the mother cell, originating pseudohyphae. Levels of magnification are as shown: Bars, 50 µm (A1 and B1) and 250 µm (A2 and B2). Results are expressed as mean (±SD) of three independent assays. * *P*<0.05, ** *P*<0.01, ***P<0.001 (C).

Of the genus *Candida*, only the species *C. albicans* and *C. dubliniensis* displays the capability to undergo dimorphic transition by producing a germ tube [6], [7]. In order to evaluate the effect of purified phlorotannins extracts in yeast dimorphic transition, and taking into account that *C. dubliniensis* doesn't produce a germ tube *in vivo*, three *C. albicans* were selected: one type strain (ATCC 10231) and two clinical isolates (D1 and D5). Purified phlorotannins extracts of species from the genus *Cystoseira* did not inhibit the germ tube formation in the tested *C. albicans* strains. However, purified phlorotannins extracts from *F. spiralis* inhibited similarly the dimorphic transition in the three *C. albicans* strains studied. In fact, more than 90% of the yeast cells treated with sublethal concentrations of purified phlorotannins extracts from *F. spiralis* (from MIC to MIC/32) presented pseudohyphae instead of germ tubes (Figure 1B1), pointing this species as a potential inhibitor of *Candida* infection dissemination [6]. MIC/32was the lowest concentration for which more than 90% of yeast cells presented pseudohyphae (Figure 1C). For MIC/64, the percentage of yeast cells with pseudohyphae was reduced for values around 70% and, for the lowest concentrations tested (MIC/128 and MIC/256), pseudohyphae represented less than 10%, and the production of germ tubes was no longer inhibited (Figure 1C).

According to these results, and in order to clarify if pseudohyphae formation could affect the adherence of *Candida* to the mucosa, an adherence assay was performed using epithelial cells. Figure 1 show the effect of purified phlorotannins extracts in the dimorphic transition of *C. albicans* ATCC 10231 and in the adherence of the yeast to epithelial cells, for the lowest *F. spiralis* concentration capable of inhibiting the dimorphic transition in more than 90% of yeast. A common distribution pattern was observed in control yeast cells, and yeast cells treated with phlorotannins. *C. albicans* treated with *F. spiralis* phlorotannins presented a widespread distribution pattern through the culture medium, with few or no adherence to epithelial cells (Figure 1B2). Contrary, in control, yeast appeared adhered to epithelial cells, and few yeast where found free in the culture medium (Figure 1A2).

### Mechanism of phlorotannins antifungal action

#### Effect on cell membrane and cell wall composition

**Figure 2:**
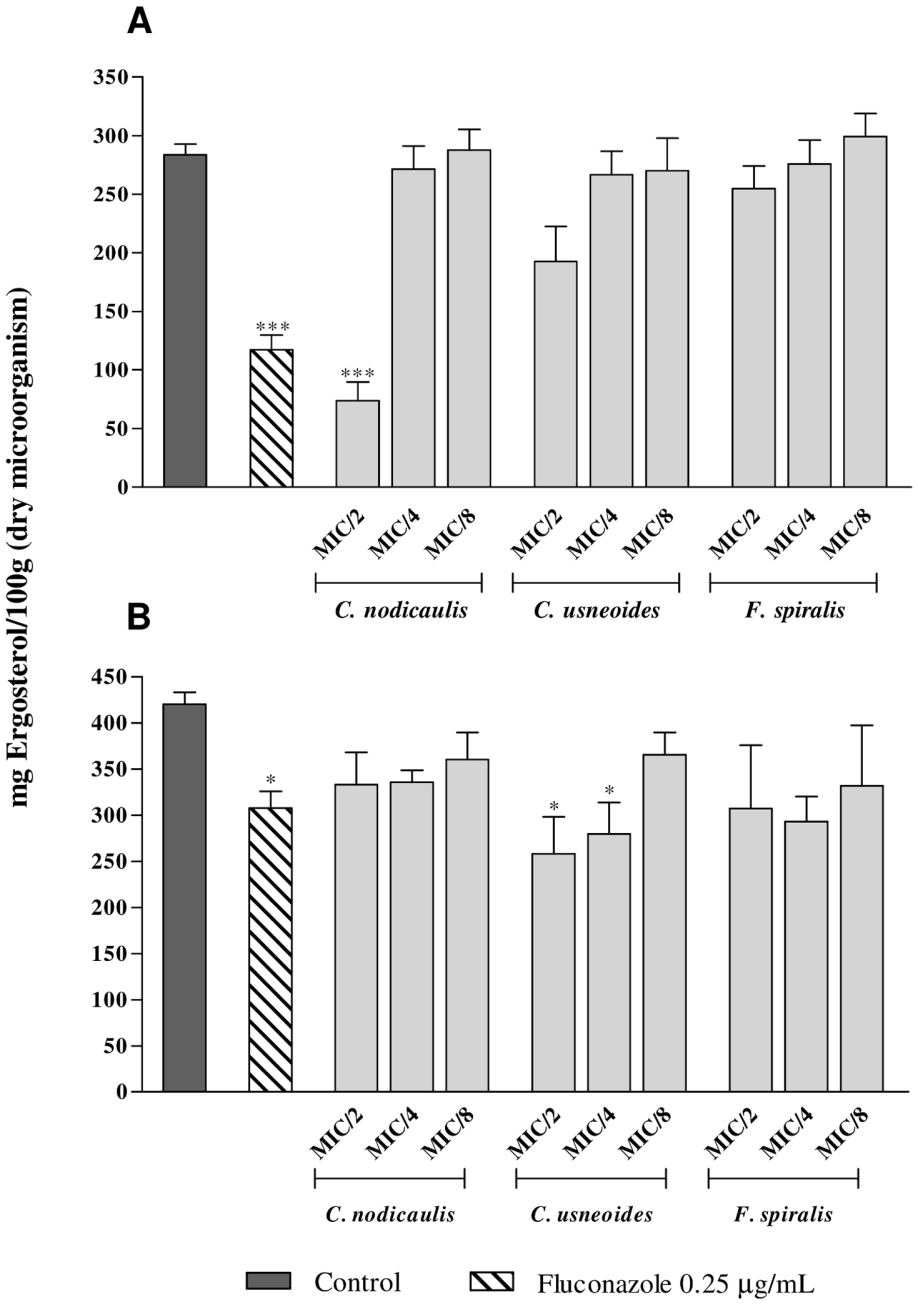
Ergosterol concentration in *C. albicans* ATCC 10231 (A) and *T. rubrum* FF5 (B) cells treated with purified phlorotannins extracts (1/2 to 1/8 of the MIC), determined by HPLC-DAD (detection wavelength 280 nm). Results are expressed as mean (±SD) of three independent assays. * *P*<0.05, ** *P*<0.01, ***P<0.001.

The effect of purified phlorotannins extracts on fungal membrane composition was evaluated by determining ergosterol by HPLC-DAD, after fungal treatment with sub-inhibitory extracts concentrations (1/2 to 1/8 of the MIC) (Figure 2). With the exception of *C. nodicaulis*, which significantly reduced the ergosterol amount in *Candida* cells (*P*<0.001), none of the remaining species significantly affected the ergosterol content of yeast cell membranes (Figure 2A). Concerning dermatophyte, only *C. usneoides* reduced ergosterol, in a similar manner to fuconazole (*P*<0.05) (Figure 2B). It was also observed that fluconazole was more effective against yeast than dermatophyte (Figure 2).

The effect of purified phlorotannins extracts on fungal cell wall composition was evaluated by measuring the amount of 1,3-β-D-glucans and chitin. 1,3-β-D-glucans levels were quantified by a fluorescence assay after microorganisms treatment with sub-inhibitory concentrations of purified phlorotannins extracts (1/2 to 1/16 of the MIC). Caspofungin, an inhibitor of glucans synthesis, was used as control. 1,3-β-D-glucans levels on cells treated with purified phlorotannins extracts were compared with those of untreated cells. None of the samples significantly affected the glucans composition of the studied microorganisms.

Chitin levels in fungal cell wall were determined spectrofotometrically. Purified phlorotannins extracts had no effect on the chitin levels of yeast cells. Regarding dermatophyte, only *F. spiralis* purified phlorotannins extracts significantly reduced the amount of chitin in *T. rubrum* (*P*<0.05).

### Screening of the respiratory chain function

**Figure 3:**
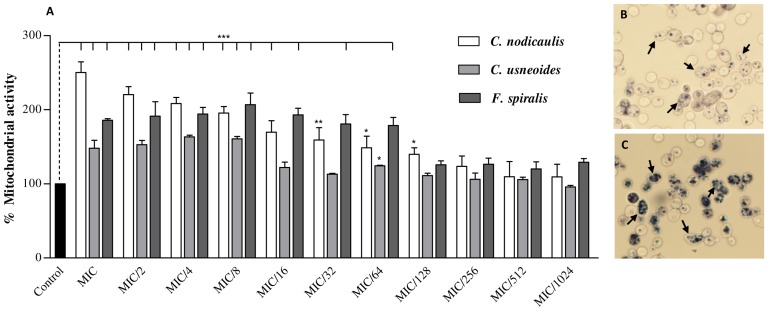
Mitochondrial activity of *C. albicans* ATCC 10231 cells treated with different concentrations of phlorotannins purified extracts. Results are expressed as the percent change of MTT reduction using the nontreated cells as control (mean (±SD) of three independent assays performed in duplicate). For concentrations lower than MIC/1024 the mitochondrial activity was similar to the untreated cells. Arrows show the formazan salts produced by *C. albicans* mitochondria. *P<0.05, **P<0.01, ***P<0.001.

In order to check whether the phlorotannins could affect the mitochondrial function, the MTT reduction assay was performed with purified phlorotannins extracts concentrations ranging from MIC to MIC/1024 (Figure 3A). Surprisingly, *Candida* cells treated with purified phlorotannins extracts presented significantly higher mitochondrial activity than the control cells, being about 2.5 times higher in cells treated with *C. nodicaulis* extract MIC (Figure 3A). With lower concentrations of purified phlorotannins extracts, the mitochondrial activity equalled the value of control cells (Figure 3A). Cells were also observed under light microscopy, and the ones treated with the extracts presented a greater density of formazan salts (Figure 3B and 3C). For *T. rubrum* spores, the MTT conversion rate decreased with increasing phlorotannins concentrations, which was the expected behaviour, and is in accordance with MIC (data not shown).

**Figure 4:**
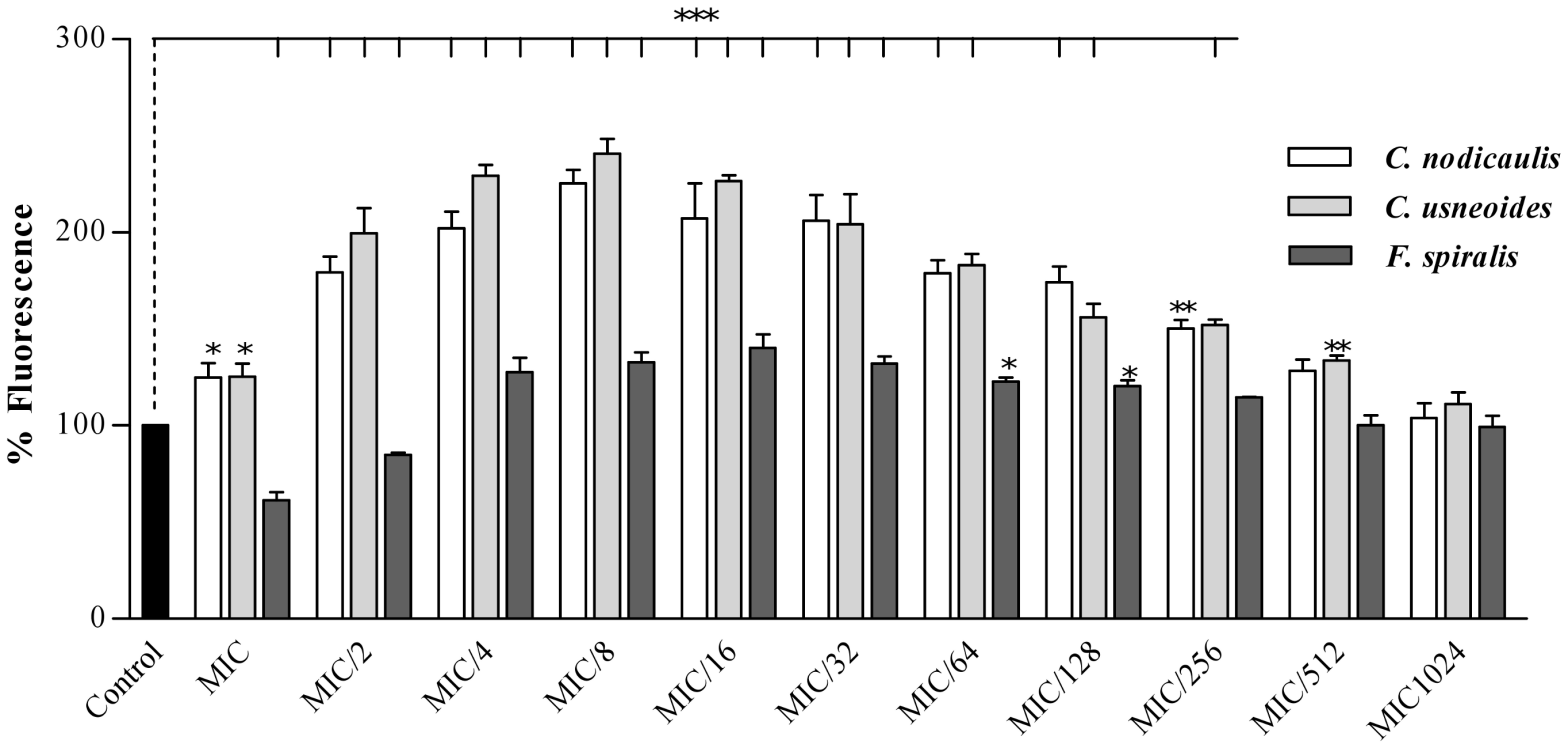
Percentage of Rhodamine 123 fluorescence of *C. albicans* ATCC 10231 cells treated with different concentrations of purified phlorotannins extracts, relative to control. For concentrations lower than MIC/1024 the percentage of fluorescence was similar to the untreated cells. Results are expressed as mean (±SD) of three independent assays performed in duplicate. * *P*<0.05, ** *P*<0.01, ***P<0.001.

Along with the MTT reduction assay, the mitochondrial membrane potential was evaluated by measuring the incorporation of the fluorescent probe RHO by *Candida* cells after treatment with purified phlorotannins extracts (Figure 4). The amount of RHO incorporated by treated cells was significantly superior to the control for almost all the tested concentrations, increasing from MIC to MIC/8 and decreasing from MIC/16 to MIC/1024 (Figure 4). The fluorescence intensity for MIC/1024 equalled the values of untreated cells.

## Discussion

*C. albicans* is a commensal yeast which commonly colonizes the mucosa of the majority of healthy humans without causing tissue damage. However, this yeast can establish disease in a variety of permissive circumstances: *Candida* cells can disseminate from mucosa and gut, being in the origin of invasive infections. Oral and vaginal thrushes are very common even in individuals with slightly weakened immunity. Biofilm-forming capacity greatly increases the potency of *Candida* to convert from the commensal stage into a virulent pathogen [1]. On the other hand, dermatophytes are keratinophilic fungi normally found growing in the dead keratinized tissue of the stratum corneum of the skin, within and around the scalp hair and in the nails. *T. rubrum* is the principal agent of onychomycosis, which are difficult treating infections due to the slow growth of nails [1]. *C. albicans* ATCC 10231 was the most sensitive of the studied yeast, and *T. rubrum* and *E. floccosum* the most sensitive of the studied dermatophytes. However only fungistatic activity was observed against yeast, purified phlorotannins extracts presented the capacity to kill the majority of the studied dermatophytes (Table 1). Thereby, these compounds can constitute promising antifungals both individually and in combined therapy.

According to the displayed antifungal activity, the purified phlorotannins extracts were object of further investigation, in an attempt to elucidate the mechanism underlying the antifungal action of these compounds. Thereby, the effect on the dimorphic transition and adherence (in *C. albicans*), on the fungal cell wall and membrane composition and on the mitochondrial function was addressed.

### Study of *C. albicans* virulence factors

#### Effect on the dimorphic transition and adherence to epithelial cells of *C. albicans*

The ability to produce a germ tube is characteristic of *C. albicans* species, being a good tool for the presumptive identification of clinical isolates [7]. The dimorphic transition of *C. albicans* is involved in the microorganism pathogenesis, its inhibition being sufficient to treat disseminated candidiasis [22], as the germ tube (Figure 1A1) is responsible for the adhesion of *Candida* cells to the mucosa, turning these infections more difficult to overcome [6].

Our results demonstrate that phlorotannins of *F. spiralis* inhibit the dimorphic transition of *Candida* cells by causing an incomplete budding, where the bud is not detached from the mother cell, originating pseudohyphae. Contrary to control cells, where germinated yeast appeared adhered to epithelial cells (Figure 1A2), yeast treated with phlorotannins from *F. spiralis* presented a uniform distribution through the culture medium (Figure 1B2). This observation confirms the adherence capacity of *C. albicans* germ tubes and the lack of that for pseudohyphae, and emphasises the potential of *F. spiralis* to reduce the virulence factor in *Candida* species. This effect can be an important target concerning the virulence of these microorganisms, as this species can significantly inhibit germination until MIC/64 (Figure 1C). Hereupon, phlorotannins of *F. spiralis* are promising to be associated with existing antifungals, possibly being beneficial for the treatment of invasive candidiasis.

### Mechanism of phlorotannins antifungal action

#### Effect on cell membrane and cell wall composition

Ergosterol is an important component of fungal cells, responsible for the maintenance of cell membrane structure and functions. Its synthesis is inhibited by azoles, which are fungistatic drugs commonly used in treatment and prevention of candidiasis [2]. The effect of purified phlorotannins extracts on fungal cell wall composition was evaluated by measuring the amount of 1,3-β-D-glucans and chitin, which are essential components of the fungal cell wall, responsible for maintaining fungal structure and normal cell growth. With the exception of *C. nodicaulis* and *C. usneoides* that significantly reduced the ergosterol amount in yeast and dermatophyte, respectively, the influence in the cell membrane composition doesn't seem to be the primary mechanism of phlorotannins antifungal action. With regard to the cell wall composition, only *F. spiralis* purified phlorotannins extracts significantly reduced the amount of chitin in *T. rubrum* (*P*<0.05), and none of the studied seaweeds affected the 1,3-β-D-glucans levels. In what concerns to chitin, it was expectable to find a much lower amount of chitin in yeast than in dermatophyte, as it constitutes only 1–10% of *Candida* cell wall in yeast form [23]. However, as this yeast undergoes dimorphic transition in RPMI medium, after the incubation period it is not in the yeast form but presenting branching filaments, for which chitin composition is similar to that of dermatophytes.

### Screening of the respiratory chain function

Unlike vertebrates, yeast respiratory chain involves more complex and flexible pathways not fully elucidated. *C. albicans* mitochondria contain three respiratory chains: the classical respiratory chain (CRC), a secondary parallel chain (PAR) and an alternative oxidative pathway (AOX). CRC capacity was verified to be twice as large as AOX while PAR capacity, which was only efficient when both CRC and AOX were blocked, represented only one tenth of the maximal oxygen consumption rate [8], [24].

A large proportion of cellular dehydrogenases, namely succinate, NADH, glycerol 3P-dehydrogenase and lactate dehydrogenases, which are part of the CRC, are responsible for the reduction of MTT to formazan salts [24], [25]. MTT reduction assay is commonly used to achieve cell viability by means of the mitochondrial function evaluation, being also used to confirm/determine MIC values. Tetrazolium salts have become some of the most widely used tools in cell biology for measuring the metabolic activity of cells ranging from mammalian to microbial origin. These salts are cleaved by mitochondrial dehydrogenases to form its purple formazan derivatives, which can be measured and reported to mitochondrial activity [25].

Face to the results (Figure 3A), and after cells observation under light microscopy (Figure 3B and 3C), we can assume a possible stimulation of cellular dehydrogenases, which presented the capacity to convert MTT at a much higher rate than the untreated cells. According to these observations, it is possible to suppose that the fungistatic activity of phlorotannins on *Candida* cells can be related to toxic effects due to increased ROS production, which follows from the respiratory rate increment [9].

During normal respiration, small amounts of toxic intermediate species are generated by partial reduction of oxygen, including superoxide, hydrogen peroxide and hydroxyl radicals. Although cells have several enzymatic (superoxide dismutase, glutathione peroxidase and catalase) and non-enzymatic (endogenous and exogenous antioxidants) systems contributing for free radicals inactivation, these molecules can accumulate and cause cell damage [9], [26]. As it is possible that purified phlorotannins extracts increase the mitochondrial respiratory rate by stimulating the activity of yeast dehydrogenases (Figure 3A and 3C), we can assume that these compounds trigger an increased production of ROS. Once the mitochondrial dehydrogenases activity on cells treated with phlorotannins purified extracts was significantly higher (*P*<0.001) than in control cells, it is possible that the cell free radical inactivation systems were not able to intercept all of the ROS formed during the respiration process, leading to toxic effects which inhibit cell division [9].

Along with the mitochondrial dehydrogenases function, the mitochondrial membrane potential is a key indicator of cellular viability. It reflects the pumping of hydrogen ions across the inner membrane during the process of electron transport and oxidative phosphorylation. RHO is a fluorescent probe commonly used for the evaluation of mitochondrial membrane potential [21]. The change in fluorescence intensity is related to the inner mitochondrial membrane depolarization, namely to the loss of membrane potential. In the absence of membrane potential, mitochondria loses the ability to sequester Ca^2+^, resulting in a loss of selectivity of the inner mitochondrial membrane and leading to a mitochondrial membrane permeability transition dependent of Ca^2+^. This change in permeability is involved in the process of injury and cell death [27], [28].

According to the results, it appears that purified phlorotannins extracts have a dual mechanism for regulating the mitochondrial membrane potential (Figure 4). The fluorescence intensity was superior to that of untreated cells for almost all extracts concentrations, leading us to assume that a hyperpolarisation state of the mitochondrial membrane could occur [29]. Although, the increase of fluorescence intensity observed from MIC to MIC/8 was more unexpected, it can be associated with cellular defence mechanisms against apoptosis [30].

Cell systems posses several proteins which are responsible for regulating programmed cell death and mitochondria integrity. In cases of severe cellular injury, anti-apoptotic proteins can block cell death by reducing the oxidative stress. The expression of these proteins is capable stabilizing the mitochondrial membrane potential [30]. Although the mechanism by which membrane polarization occurs is still obscure, it is known that the loss of mitochondrial membrane potential constitutes an early event during apoptosis in some systems [31]–[33]. As so, a biphasic change in membrane potential can occur for higher concentrations of tested compounds, with an early hyperpolarization followed by a depolarization [32].

According to this, it could be hypothesised that at the highest concentration (MIC) there is increased expression of anti-apoptotic proteins by the cell, in a tentative to resist apoptosis, and therefore a quicker stabilization of the membrane potential takes place. As the concentration of phlorotannins decreases (from MIC to MIC/8), the cellular injury and the expression of anti-apoptotic proteins lowers, so that the membrane potential is not established as soon.

The second mechanism by which phlorotannins seem to affect the membrane potential is not probably related to anti-apoptotic defences. The accumulation of ROS inside cells can lead to alterations in cell membranes permeability, leading to the increase of cytosolic Ca^2+^ concentrations, both by the release of Ca^2+^ from the intracellular stores and by the increased influx over the cytoplasm membrane [9]. As soon as the phlorotannins concentration lowers (from MIC/8 to MIC/1024), the membrane potential may be gradually replaced. The cell recovers from the hyperpolarization state, the mitochondrion recovers the membrane potential, and the fluorescence values equal the control.

Taking into account such hypotheses, there seem to be two possible mechanisms to explain the behaviour of mitochondria in *C. albicans* ATCC 10231 when exposed to phlorotannins. The mechanism taking place for higher phlorotannins concentrations (from MIC to MIC/8) can be regulated by anti-apoptotic proteins expression, once cells are undergoing a major aggression that puts at risk their viability. For lower phlorotannins concentrations (from MIC/16 to MIC/1024) cellular aggression will not be sufficient to trigger anti-apoptotic defence mechanisms, and a mechanism related to the change in Ca^2+^ permeability can take place.

In conclusion, this work evidences the antifungal capacity of phlorotannins against a wide range of yeast and filamentous fungi. In a general way, *C. nodicaulis* revealed to be the most effective species against the studied fungi. While not being lethal for *Candida* species under the tested concentrations, this seaweed presented the lowest MIC/MLC for both yeast and dermatophytes, also being lethal for the last ones. Although the mechanism of action of these compounds was not completely elucidated, there are evidences pointing to some effect on ergosterol and chitin composition in filamentous fungi, and ergosterol and respiration in yeast. Nevertheless, the main goal of phlorotannins seems to be its capacity to reduce the virulence factor in *C. albicans*, by inhibiting the dimorphic transition.

The hyperactivity of mitochondrial dehydrogenases caused by phlorotannins can lead to the accumulation of ROS and Ca^2+^, which in turn may result in membrane hyperpolarization, incompatible with normal cell metabolism. Nevertheless, the mechanism of action of these compounds needs further investigation, namely in what concerns to their action over some important cell enzymes like proteases, lipases and α-glucosidase, which play an important role in cell metabolism [34], [35].

The increment in combining antifungal medications with different mechanisms of action can lead to better therapeutic responses. Contrary to what happens with some commercially available antifungal drugs, the effect on yeast mitochondria activity can be the primary mechanism of action of phlorotannins. Thus, the challenge remains to associate compounds from natural matrices with existing antifungal drugs for which there is some resistance. There are some data indicating that phlorotannins do not act similarly in the mitochondria of mammalian and yeast [12]. In this sense, searching for differences between mammalian and fungi mitochondria, in the classical and alternative components of the mitochondrial respiratory chain, may provide new potential therapeutic targets in treating pathogenic fungal infections.
